# Familial Pulmonary Capillary Hemangiomatosis Early in Life

**DOI:** 10.1155/2011/827591

**Published:** 2011-12-19

**Authors:** Johannes Wirbelauer, Helge Hebestreit, Alexander Marx, Eugene J. Mark, Christian P. Speer

**Affiliations:** ^1^University Children's Hospital, University of Wuerzburg, Josef-Schneider-Straße 2, 97080 Wuerzburg, Germany; ^2^Department of Pathology, Faculty of Medicine Mannheim, University of Heidelberg, 68167 Mannheim, Germany; ^3^Harvard Medical School, Department of Pathology, Massachusetts General Hospital, Boston, MA 02114, USA

## Abstract

*Background*. Pulmonary capillary hemangiomatosis (PCH) is a rare disease, especially in infancy. Four infants have been reported up to the age of 12 months. So far, no familial patients are observed at this age. *Patients*. We report three siblings, two female newborns and a foetus of 15-week gestation of unrelated, healthy parents suffering from histologically proven PCH. The first girl presented with increased O_2_ requirements shortly after birth and patent *ductus arteriosus* (PDA). She subsequently developed progressive respiratory failure and pulmonary hypertension and died at the age of five months. The second girl presented with clinical signs of bronchial obstruction at the age of three months. The work-up showed a PDA—which was surgically closed—pulmonary hypertension, and bronchial wall instability with stenosis of the left main bronchus. Transient oxygen therapy was required with viral infections. The girl is now six years old and clinically stable without additional O_2_ requirements. Failure to thrive during infancy and a somewhat delayed development may be the consequence of the disease itself but also could be attributed to repeated episodes of respiratory failure and a long-term systemic steroid therapy. The third pregnancy ended as spontaneous abortion. The foetus showed histological signs of PCH. *Conclusion*. Despite the differences in clinical course, the trias of PCH, PDA, and pulmonary hypertension in the two life born girls suggests a genetic background.

## 1. Introduction

Pulmonary capillary hemangiomatosis (PCH) is a rare pulmonary disease characterized by numerous capillary-sized blood vessels that proliferate diffusely throughout the pulmonary interstitial tissue, pulmonary blood vessels, and airways [1]. The Venice Clinical Classification of Pulmonary Hypertension displays both PCH and pulmonary veno-occlusive disease as pulmonary hypertension associated with significant venous or capillary involvement [2]. Since the first description by Wagenvoort et al. in 1978 [3], PCH was diagnosed in individuals of different ages ranging from neonates to the elderly [4]. In a series of 37 patients with PCH only four were identified up to an age of 7 years [5]. Congenital PCH has been reported in two additional patients [6]. One was a male twin, stillborn after 30 weeks of gestation. Autopsy revealed absent kidneys in addition to PCH. The diamniotic, monochorionic twin was alive and well. Another male newborn presented with severe mitral regurgitation and pulmonary hypertension requiring cardiac surgery and died at the age of 9 weeks. PCH was diagnosed post mortem. To our knowledge only one familial occurrence in three siblings has been reported, who all died because of PCH and pulmonary hypertension in young adulthood [7]. None of them had additional anomalies or malformations. We report for the first time three siblings, two female term newborns and a foetus stillborn at 15 weeks of gestation, with histologically proven PCH. Both newborns had patent ductus arteriosus (PDA) and pulmonary hypertension (PH) which manifested in the neonatal period and in early infancy, respectively.

## 2. Patients

### 2.1. Patient 1

**Figure 1:**
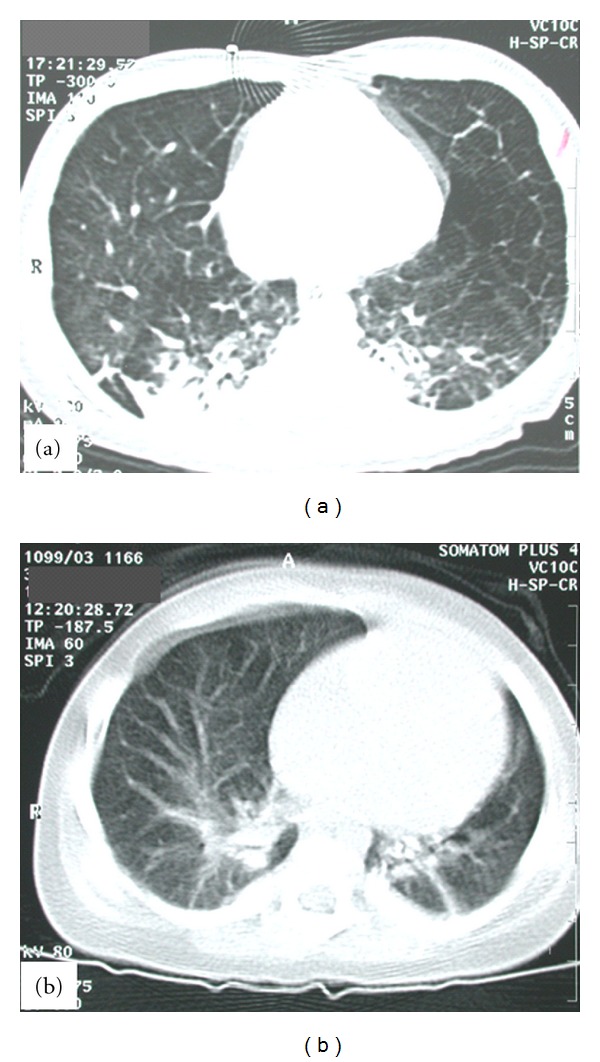
Computed tomography (CT) of the thorax. (a) CT of the thorax of patient 1 at the age of 3.5 months. Small cystic, emphysematous transformations ubiquitously involving both lungs and consolidations in the dorsal parts of the lungs were found. (b) CT of the thorax of patient 2 at the age of 4 months. Consolidations in both lungs with some cystic areas, a relatively small left lung, and enlarged central pulmonary arteries with rarefication of the branches in the periphery are shown. Please note the more focal involvement of the lungs compared to patient 1.

**Figure 2:**
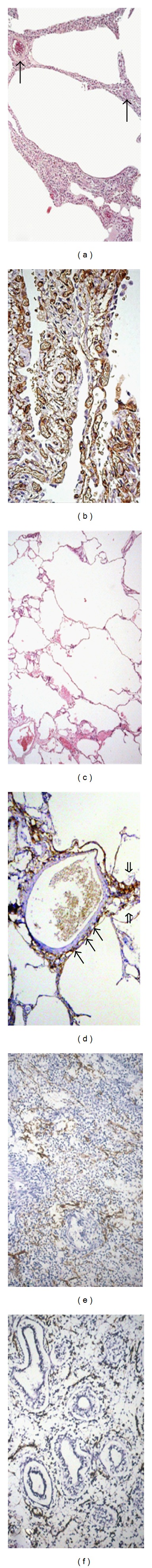
Lung biopsies of patient 1 (a and b), patient 2 (c and d), patient 3 (e), and control (f). (a) Altered lung architecture with slight emphysema and slight to moderate thickening of alveolar walls due to proliferation of capillaries. Note venous sclerosis (→) and the capillary duplications in some alveolar walls. Changes reflect pulmonary arterial hypertension (Hematoxylin and Eosin, ×100), (b) High-power view of thickened alveolar septa due to proliferation and tortuosity of capillaries. Endothelial cells lining the inner surface of capillaries are marked by CD-34 staining (Immunopoeroxidase, ×200), (c) Irregular emphysematous changes and focal capillary proliferation in thickend alveolar walls. Changes are less prominent than in patient 1 (Hematoxylin and Eosin, ×100), (d) Capillary proliferation and protrusion of tufts of capillaries is found in some alveolar septa (*⇒*) and in the wall of a vein (→). Endothelial cells lining the inner surface of capillaries are marked by CD-31 staining (Imminoperoxidase, ×200), (e and f) Post mortem lung histology of patient 3 (e) versus control (f). pseudoglandular stage of lung development at 15 weeks of gestational age. Endothelial cells express CD-31. (Immunoperoxidase, ×200), (e) Broadened space between pseudoglands with proliferation of capillaries in lung tissue of patient 3. Regressive changes are due to advanced autolysis following intrauterine death, (f) Normal lung architecture at 15 weeks of gestational age.

The girl was born after an uneventful pregnancy at 42 weeks of gestation as first child of unrelated, healthy parents. The 28-year-old mother reported no prior pregnancies, the birth weight was 2860 g (7th centile), Apgar scores after 5 and 10 minutes were 6 and 8, respectively, and the umbilical artery pH was 7.18. The newborn presented directly after birth with an increased O_2_-requirement (fraction of inspired oxygen (FiO_2_) of 0.4) and need for continuous positive airway pressure (CPAP). Chest radiograph showed an enlarged heart silhouette with signs of increased pulmonary blood flow. A hemodynamic significant PDA was closed surgically at the age of 18 days after an unsuccessful attempt with indomethacin. However, increased oxygen requirement persisted. Echocardiography suggested that PH and cardiac catheterization showed systemic blood pressure in the pulmonary arteries at rest and suprasystemic levels during activity. Pulmonary vein obstruction was excluded. At the age of 10 weeks, the girl acquired viral pneumonia and mechanical ventilation became necessary. Chest radiography showed a seriously enlarged heart-silhouette with cystic and consolidated areas of the lung. Computed tomography (CT) of the thorax demonstrated small cystic, emphysematous transformations of both lungs (Figure 1(a)). Histological examination of an open lung biopsy performed at the age of 14 weeks showed ectatic alveoli (Figures 2(a) and 2(b)). These findings were interpreted as cystic adenomatoid malformation Stocker type III, and the case was subsequently published because of the unusual combination of this pulmonary condition with pulmonary hypertension [8]. Only the reevaluation of tissue samples together with the lung biopsy of her sister (patient 2, see) could establish the diagnosis of PCH in this patient. 

Global respiratory insufficiency developed after the age of 3 to 4 months. Radiologically, the areas of the lung affected by abnormal tissue increased. Despite the use of different ventilatory strategies, nitric oxide and iloprost inhalation, the patient died from cardiorespiratory failure due to a viral pneumonia at the age of 5 months.

### 2.2. Patient 2

This girl was born after 38 weeks of gestation. Pregnancy and delivery were unremarkable, the birth weight was normal (3440 g; 60th centile), and Apgar scores were 10 after 5 and 10 minutes, respectively. On physical examination, mild anomalies were present such as an umbilical hernia, a diastasis of the *Musculus rectus abdominis*, and a high arched palate. However, the girl was otherwise asymptomatic until she developed the clinical picture of an obstructive bronchitis at the age of 3 months. While the initial chest X-ray after birth was normal, chest radiography and CT of the thorax at the age of 4 months showed consolidations in both lungs with some cystic areas, a relatively small left lung, and enlarged central pulmonary arteries with rarefication of the branches in the periphery (Figure 1(b)). Bronchoscopy demonstrated subtotal stenosis of the left main malacic bronchus and bronchomalacia in other bronchi. However, a hemangioma in the left main bronchus could not be excluded. Repeated echocardiography showed a PDA without hemodynamic significance, a remarkably long intramural course of the right coronary artery, and—during follow-up—increasing enlargement of the right ventricle with thickening of the right ventricular wall. Pulmonary pressure could only sporadically be estimated by echocardiography, but was increased at the age of 4 months and thereafter to nearly half of systemic arterial pressure. PDA was surgically closed at the age of 7 months. Open lung biopsies were taken twice, one from the right upper lobe during the PDA-operation and another from the left lung at the age of 12 months. While the first biopsy showed only minor changes suggestive of PCH, the second biopsy confirmed this diagnosis (Figures 2(c) and 2(d)). 

**Figure 3:**
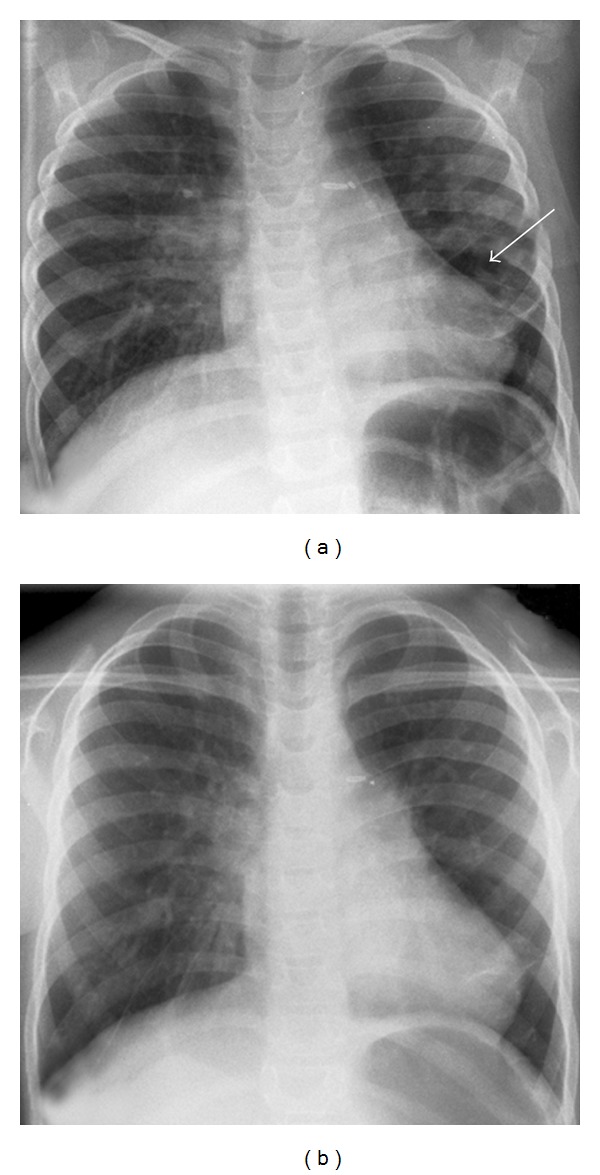
Chest X-ray of patient 2 in anterior-posterior condition, (a) Chest X-ray at the age of 3 years: there are signs of hyperlucent and hyperinflation with cystic-imaging areas (→) and laterally ascending ribs as a sign of lung inflation. In some areas of the lungs, there are consolidations. The heart silhouette is enlarged and clips are present in typical location after surgical closure of a patent ductus arteriosus, (b) Chest X-ray at the age of 5.5 years: persistent signs of bronchial obstruction. Chronic consolidations are found perihilar at the right side and behind the heart.

The clinical course of patient 2 was remarkably different compared to that of her deceased sister (Table 1). Patient 2 presented mainly with wheezing and episodes of respiratory distress leading to hospital treatment first time after 3 months of age. Episodes of increased oxygen requirements occurred only during viral respiratory infections. Symptoms responded relatively well to inhalations with salbutamol and ipratropium-bromide. However, systemic steroids were necessary over prolonged periods of time with good effects. Except for elective surgery, the girl was never on mechanical ventilation.  Figure 3(a) shows the chest X-ray at the age of three years. A recent X-ray of the thorax is shown in Figure 3(b) demonstrating focal chronic consolidations and bronchial obstruction. Growth and weight gain of patient 2 were blunted during the first years of life, but height and weight are now, at the age of seven years, at the 12th and 56th centile, respectively. There is a persisting retardation in motor and speech development. However, the girl continuously improves her skills and was able to visit a regular kindergarten. At the moment she visits a specialized school for physically handicapped children because of reduced cardiopulmonary capacity limiting even everyday activities. The girl does not need supplemental oxygen due common colds since her sixth year of life.

### 2.3. Patient 3

After an initially unremarkable pregnancy, intrauterine death was diagnosed at 15 weeks of gestation and spontaneous abortion occurred. Histological examination of the fetal lung showed abnormal capillaries (Figures 2(e) and 2(f)). No other abnormalities were diagnosed. The cause for the stillbirth remained unclear.

## 3. Discussion

To our knowledge, we report the first cases of familial, congenital PCH with clinical manifestation in the neonatal period or early infancy, respectively. While the pulmonary disease was evident already at birth in patient 1, patient 2 became symptomatic only after three months. PCH was established in both patients by histological and immunohistological examination of lung tissue. Changes associated with PCH were not different from those reported in other patients with PCH [1, 3].

As in patient 1 and also in patient 2, PCH is often associated with PH [9]. Leading clinical symptoms like pulmonary distress and pulmonary hypertension in PCH have to be differentiated from other diseases like hematologic or immunologic diseases [10]. Interestingly, some molecular findings suggest that pulmonary veno-occlusive disease (PVOD) and PCH might represent different phenotypes of the same disease [2]. It has been suggested that capillary growth in and around the pulmonary vessels is involved in the pathogenesis of PH in PCH [11]. It is, however, unknown whether a dysregulation in molecular mechanisms of pulmonary vascular development is responsible for PCH [12]. Most likely, it develops secondary to a reactive veno-occlusive phenomeneon in combination with recurrent pulmonary hemorrhage, thrombosis, and infarction [13, 14]. Mechanical intravascular hemolysis was reported in one patient with PCH [5]. Increased platelet-derived growth-factor and growth-factor receptor gene expression may play a role in the pathogenesis of PCH [15]. One might speculate that acquired disorders of the pulmonary capillary and venous structure more often present as PVOD. However, the genetic background of PCH is still not understood. A possible impact of genetic factors is further supported by the familial PCH reported formerly [7] and in our patients. Recently, genetic deletions of the FOX gene cluster were found in alveolar capillary dysplasia with misalignment of pulmonary veins, another disorder of pulmonary blood vessels [16].

While there might be some similarities to vascular neoplasms in adults, the few children with PCH reported under the age of 6 months presented with severe malformations of the cardiovascular or renal system [6]. Based on the familial occurrence in our patients, we speculate that PCH in at least some patients with the congenital or infantile form of the disease could have a genetic background. 

Despite histological similarities in patients 1 and 2, the clinical course was remarkably different, possibly reflecting a nearly complete involvement of the lungs in patient 1 but only focal distribution of hemangiomatosis in patient 2. The diagnosis of PCH in neither patient could be made by chest X-ray. More sensitive but not specific was the imaging of the lungs by CT of the thorax in agreement with previous studies [17, 18]. Though lymph node pathology has been described in PCH, lymphatic congestion or lymph node hyperplasia was not evident in our cases [19]. Differential diagnosis to be considered in case of pulmonary hypertension and the observed CT changes may include idiopathic pulmonary arterial hypertension, PVOD, pulmonary interstitial fibrosis, micro-thrombo-embolic pulmonary vascular disease, pulmonary lymphangiomatosis [20], and cystic adenomatoid lung malformation. Histological examination was required to establish the diagnosis of PCH in our patients. Though emphysematous changes in PCH of adults are missing, these emphysematous changes seem to be typical for familial cases. An increased risk of severe bleeding during biopsy was reported recently [21], but noninvasive diagnostic in our cases was not conclusive. As mentioned earlier, the clear differentiation of PCH from cystic adenomatoid lung malformation was difficult in patient 1 [8] because there was absence of bronchial cartilage with multiple small cysts in all segments of the lung. In retrospect staining for vasculature could clearly identify the changes typical for PCH with massive increase in capillaries infiltrating alveolar septa and the walls of vessels.

The age of manifestation and the clinical signs differ largely between the patients reported in the literature [1, 3, 6, 7, 15]. In our patients presentation occurred in fetal and neonatal time and infancy, respectively. A further 30-weeks-old stillborn preterm infant with PCH associated with absent kidneys has been reported [6]. In addition, a term newborn of 38 weeks' gestation with PCH associated with severe mitral regurgitation and endocardial fibrosis died at the age of 9 weeks [6]. Some patients with PCH manifestation in childhood have been described [5, 22]. However, most patients with PCH are diagnosed at the age of 20–40 years [22]. Clinical features also differ among patients. Although two thirds of all patients primarily suffer from signs of dyspnea [5], hemoptysis is described as first clinical symptom as well [23, 24]. X-ray findings are not specific and CT-scans of the thorax usually show nonspecific alterations [5]. Therefore, PCH is frequently misdiagnosed ante mortem when biopsy is not performed. Diagnosis of PCH might be substantiated by bronchoalveolar lavage and—in older children and adults—by assessing CO diffusing capacity [5, 21]. Right ventricular hypertrophy can be detected by electrocardiography, because progressing disease results in pulmonary hypertension with poor prognosis. PCH is frequently misdiagnosed ante mortem. Though it is known that open lung biopsy can result in massive hemorrhage in some cases, this invasive procedure has to be considered in patients with pulmonary hypertension of unknown aetiology to eventually prove a specific diagnosis. In our two patients, biopsy was performed without adverse side effects.

Due to the very low incidence of PCH, no therapeutic regimens have been defined so far. Therapeutic strategies like iv-epoprostenol to lower PH may produce pulmonary edema in patients with PCH and are therefore contraindicated [5, 21]. Because of the malgenesis of pulmonary vessels, steroids and cyclophosphamide were used, but also given unsuccessfully in some patients [22]. In contrast our patient 2 seemed to benefit from systemic steroids at least clinically in the short run. *α*-Interferon was reported to be effective in one patient [5, 25, 26]. Experimental use of the antibiotic drug doxycycline as an inhibitor of angiogenesis was successful in another patient [23]. In adults, lung-transplantation can be offered [2, 21]. With the exception of systemic steroids, we did not use any of the above mentioned therapeutic strategies. 

## 4. Conclusion

Our patients show that familial PCH might occur early in life. The rare disorder was not associated with other life-threatening pathology but with signs of pulmonary hypertension and a persistent patent ductus arteriosus. Thus, the diagnosis should be considered in young children with right heart failure, right ventricular hypertrophy, or pulmonary hypertension. Open lung biopsy was required to confirm the diagnosis. There is no established therapy for PCH early in infancy, but our patients demonstrate that outcome may be quite variable, even within a family. Despite the differences in clinical course, the trias of PCH, PDA, and pulmonary hypertension in the two life born girls suggests a genetic background.

## Figures and Tables

**Table 1:** Clinical characteristics of the 3 patients with PCH.

	Patient 1	Patient 2	Patient 3
Gestational age (wks.)	42	38	15
Malformations	—	Umbilical hernia diastasis m. recti gothic palate	—
Onset of symptoms	1st day of life	3 months	—
Pulmonary symptoms	Supplemental oxygen need since birth	Recurrent obstructive bronchitis in infancy	—
Patent ductus arteriosus	Surgical closure in neonatal period	Surgical closure at the age of 7 months	Prenatal status
Mechanical ventilation	Ventilated for almost entire life span	Only used for elective surgery	—
Pulmonary hypertension	Confirmed during heart catheterization	Suspected by echocardiography and electrocardiography	—
Outcome	Death at the age of 5 months due to respiratory failure	Poor growth during first years recurrent obstructive bronchitis neuro-developmental delay	Abortion
